# Understanding the ADHD-Gut Axis by Metabolic Network Analysis

**DOI:** 10.3390/metabo13050592

**Published:** 2023-04-26

**Authors:** Ezgi Taş, Kutlu O. Ülgen

**Affiliations:** Department of Chemical Engineering, Bogazici University, Istanbul 34342, Turkey; ezgi.tas@boun.edu.tr

**Keywords:** gut microbiota, ADHD, short chain fatty acids, neurotransmitter precursors, probiotic

## Abstract

Attention deficit hyperactivity disorder (ADHD) is a neurodevelopmental disorder diagnosed with hyperactivity, impulsivity, and a lack of attention inconsistent with the patient’s development level. The fact that people with ADHD frequently experience gastrointestinal (GI) dysfunction highlights the possibility that the gut microbiome may play a role in this condition. The proposed research aims to determine a biomarker for ADHD by reconstructing a model of the gut-microbial community. Genome-scale metabolic models (GEM) considering the relationship between gene-protein-reaction associations are used to simulate metabolic activities in organisms of gut. The production rates of dopamine and serotonin precursors and the key short chain fatty acids which affect the health status are determined under three diets (Western, Atkins’, Vegan) and compared with those of healthy people. Elasticities are calculated to understand the sensitivity of exchange fluxes to changes in diet and bacterial abundance at the species level. The presence of Bacillota (genus *Coprococcus* and *Subdoligranulum*), Actinobacteria (genus *Collinsella*), Bacteroidetes (genus *Bacteroides*), and Bacteroidota (genus *Alistipes*) may be possible gut microbiota indicators of ADHD. This type of modeling approach taking microbial genome-environment interactions into account helps us understand the gastrointestinal mechanisms behind ADHD, and establish a path to improve the quality of life of ADHD patients.

## 1. Introduction

Attention deficit hyperactivity disorder (ADHD) is a neurodevelopmental disorder diagnosed with hyperactivity, impulsivity, and lack of attention inconsistent with the patient’s development level [1]. Today, ADHD is one of the most common children’s psychiatric disorders [2] and it is usually continuous throughout life [3]. Up to 85% of diagnosed patients report that the symptoms continue during adolescence and 60% of them point out that ADHD affected their adult life too [4,5]. The relationship between biological gender and the incidence of the disorder is around 1:5 to 1:9 in girls and boys respectively [6]. ADHD diagnosis depends on psychological evaluation, there is neither a blood test nor other physical tests [7]. There are two types of diagnostic criteria for ADHD—inattention and hyperactivity/impulsivity—but a biomarker has not been identified yet. There are possible risk factors, e.g., birth weight below 1.5 kg increases the risk of ADHD two to three times but not all children with low birth weight have ADHD [1]. Environmental factors during the prenatal period such as maternal smoking, alcohol consumption, heavy metal exposure, and nutrition habits, and toxin exposure after birth, may cause ADHD [8]. There is also a genetic predisposition to this disorder, that is children who have parents and/or siblings with ADHD are at high risk.

There is a bidirectional relationship between the brain and the gut, called the gut-brain axis [9]. This axis is responsible for cognitive functions like mood and motivation [10,11]. The mechanism of brain-gut axis communication contains neuro-immuno-endocrine mediators [12]. In terms of communication networks, the central nervous system (CNS), autonomic nervous system (ANS), enteric nervous system (ENS), and hypothalamic pituitary adrenal (HPA) axis participate in the gut-brain axis [13]. Recent studies showed that some neuroactive molecules like gamma-aminobutyric acid (GABA), serotonin, catecholamines like dopamine and norepinephrine (noradrenaline), acetylcholine, and histamine are produced by gut microbiota and influence the gut-brain axis network (Table 1). This interaction might be a result of an intestinal barrier modulation [13]. Gut microbiota affects gut permeability, and different metabolic cycles, such as carbohydrate metabolism and immunity.

Carbohydrate metabolism in the human gut results in short-chain fatty acid and gas production [14]. Acetate, butyrate, and propionate have the highest abundance in the human intestine. The ratio between them is 3:1:1, and butyrate is considered the most important short chain fatty acid for a healthy metabolism [14,15]. There are several bacteria responsible for acetate production, but butyrate and propionate-producing bacteria are more specific [16,17]. Firmicutes bacteria, such as some Lachnospiraceae family and species *Faecalibacterium prausnitzii*, are the major butyrate producers in the human intestine [14,18]. Propionate production is mostly handled by Bacteroides with the help of *Negativicutes* and *Clostridium* bacteria [14]. SCFAs have a variety of advantageous effects on several facets of human energy metabolism; however, our knowledge of the underlying molecular pathways is still limited. The lack of information regarding the actual fluxes of SCFAs and the metabolic processes, which SCFAs regulate, has so far hampered the field.

The composition of the gut microbiome depends mostly on dietary habits. Drug use, nicotine and alcohol consumption, and stress also alter the gut microbiome [19]. These changes (dysbiosis) are associated with several health problems such as irritable bowel syndrome (IBS) [20], autism spectrum disorder (ASD) [21], depression, and anxiety [22]. The gut microbiota is also known to affect behavioral symptoms in neurodevelopmental diseases. A Dutch sample of 42 adolescents and young adults with ADHD revealed a significant increase in a genus from the family Ruminococcaceae, which was associated with inattention symptoms [23]. *Prevotella* and *Alistipes* species as well as *Akkermansia muciniphila*, a novel mucin-degrading bacterium, have been reported as the most prevalent in ASD children [24,25,26]. Decreased levels of some health-promoting bacteria (e.g., *Bifidobacterium*) and metabolites (e.g., FAA, SCFA) were also reported in autistic children [24]. Due to their anti-inflammatory effects on the central nervous system, SCFA-producing bacteria have been demonstrated to potentially contribute to ADHD and autism through a number of gut-brain pathways [27]. Short-chain fatty acid (SCFA) producers include *Prevotella* species and some *Coprococcus* species. Thus, children with ADHD had lower levels of the genus *Prevotella* than did controls [27]. Some bacterial species are more active in the production of SCFAs, such as *Bacteroides* spp. and *Clostridiae* spp. [23]. *Faecalibacterium* and *Ruminococcus* and *Bifidobacterium* genera were positively correlated with total SCFA [24]. High levels of SCFA worsened the symptoms of autistic individuals [28,29]. Propionic acid showed neurobiological effects in test animals such as rats [24,30]. At present, there are contradictory conclusions in the literature regarding the role of the microbiome in neuropsychiatric disorders.

**Table 1:** Neurochemical compounds and genus relationship [31].

Neurotransmitter Precursors Found in the Human GI Tract	Neurotransmitters	Genus	References
L-Glutamate	GABA	*Lactobacillus*, *Bifidobacterium*, *Bacteroides*, *Parabacteroides*, *Escherichia*	[32,33]
L-tryptophan	Serotonin	*Streptococcus*, *Escherichia*, *Enterococcus*, *Lactococcus*, *Lactobacillus*	[34,35]
L-tyrosine, Phenylalanine	Norepinephrine	*Escherichia*, *Bacillus*	[35,36]
L-tyrosine, Phenylalanine	Dopamine	*Escherichia*, *Bacillus*, *Lactococcus*, *Lactobacillus*, *Streptococcus*	[34,35,36]
Choline	Acetylcholine	*Lactobacillus*, *Bacillus*	[37,38,39,40]
L-histidine	Histamine	*Lactobacillus*, *Lactococcus*, *Streptococcus*, *Enterococcus*	[41,42,43]

The lack of deep information on the brain-gut axis for ADHD individuals leads us to investigate gastrointestinal reasons (gut metabolic products) by reconstructing a model of the gut-microbial community with the ultimate aim of determining a biomarker for ADHD. Community growth and elasticities are predicted to understand the sensitivity of exchange fluxes to changes in diet (Western, Atkins’, and Vegan) and bacterial abundance at the species level. This type of modeling approach taking microbial genome–environment interactions into account may help us understand the gastrointestinal mechanisms behind ADHD and offer prebiotic/probiotic treatment options, establishing a path to improve the quality of life of ADHD patients.

## 2. Materials and Methods

### 2.1. Data

The work of Aarts et al. [44] provide bacterial abundance data in the gut of adolescents with ADHD, containing 19 people diagnosed with ADHD and 77 healthy people, 96 samples in total. The average age of ADHD subjects is 19.5 with a standard deviation of 2.5. On the other hand, the mean age of control subjects is 27.1 but the standard deviation in the group is 14.3 years which is significant. In addition, while 53.2% of the Control group population is male, this ratio raises to 68.4% for ADHD subjects. 25% of the people in the Control group were siblings of ADHD patients. The unaffected siblings of ADHD cases (21/77), perhaps living in the same home and eating comparable foods, are analyzed here, i.e., only the matching subjects (pairs) are considered as given in Table 2. In the end, 20 samples out of 96 samples were determined to be usable. The dataset is handled according to age and gender differences.

### 2.2. Reconstruction of Community Model

**Figure 1:**
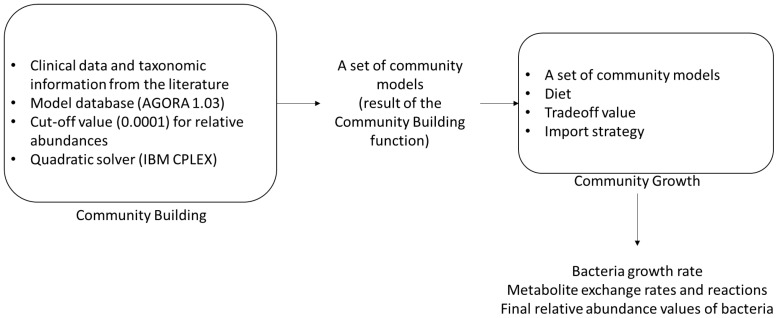
Illustration of the research method.

Using the bacterial abundance data retrieved from Aarts et al. [44], we collected the organisms from AGORA database [45], which comprises manually curated metabolic models of more than 7000 strains, including those of the human gut microbiome. MICOM software is employed for the reconstruction of the gut community model. MICOM is based on the COBRApy Python package for constraint-based modeling of biological networks [46]. By MICOM, the species-specific genome scale metabolic models are combined to create a unified community model that simultaneously takes into account the exchange fluxes between individuals and between individuals and their environment. To determine the optimum growth of the gut microbiota, the cooperative trade-off technique in MICOM is employed and the objective function is selected as the maximization of microbial community biomass growth. The relative abundances of the different species are used to account for the community composition during the simulations, and the cutoff value for bacterial abundance is selected as 10^−4^. The most feasible growth rate for the gut community model is obtained by a tradeoff value of 0.7. During community growth, the lowest possible metabolite intake is aimed at due to the possible lack of resources. IBM CPLEX [47] is used as the optimization solver (quadratic solver) in all the model reconstructions and analyses, and the cutoff value is selected as 0.0001 (Figure 1).

The flux balance analysis is performed by MICOM. By assuming that all fluxes in the biological system are in a steady state and maximizing a biomass reaction that is specific to the organism, flux balance analysis can get approximations of the fluxes for a certain organism. This can be expressed as a constrained linear programming problem for the fluxes, *v_i_* (in millimoles per gram dry weight per hour) using the stoichiometric matrix *S*., which has metabolites in its rows and reactions in its columns. Typically, the biomass reaction *v_bm_* is normalized so that it will yield 1 g of biomass, which corresponds to a unit 1/h representing the organism’s growth rate. The upper and lower boundaries provide thermodynamic restrictions to the fluxes or limit interactions with the environment, i.e., exchange fluxes. Each organism is typically embedded in an external compartment (the gut lumen) that represents the community environment, in order to depict a community model that contains several species with different abundances (in grams dry weight) for each organism. With the use of MICOM, a flux balance analysis is carried out by adding exchanges for the environment compartment and exchanges between a specific organism and the environment. The input for MICOM is abundances, which it uses to forecast growth rates and fluxes. The gut metabolic community model includes 58 microbial species and 968 exchange metabolites with their exchange reactions showing the overall metabolic capacity of the microbial community.

The reconstructed gut community model is introduced to different diets to observe how the bacterial community in the gut will react to different external factors. Western, Atkins’, and Vegan diets are selected as the community growth media. Western Diet represents a typical American diet and is retrieved from the VMH (virtual metabolic human) database [48] as well as from MICOM documents. Atkins’ Diet is derived from the high fat, low carb diet given in VMH [49]. The amount of energy provided by the diet, as defined in VMH, consists of 1.7% carbohydrates, 70% lipids, and 24% proteins [50]. Vegan Diet is a plant-based diet, with any type of food of animal origin removed from it. The energy source distribution of the Vegan Diet is 15.66% proteins, 20.99% lipids, and 63.35% carbohydrates, as given in VMH [51]. After community building, the analyses include community growth, tradeoff, and elasticities. The impact of targeted interventions on the net consumption or production of SCFAs as well as neurotransmitter precursors by the microbiota is predicted.

### 2.3. Diversity Analyses

Two diversity measures, Alpha and Beta diversities, are here used to characterize communities quantitatively by using the number of species and the number of individuals on any level, sample or community. The first compartment of alpha diversity is the indices of species richness, also known as variety indices. The species richness index is defined as the total number of species, S, in the sample. Among the several indices used to determine diversity in the sample, Chao’s index, Shannon’s index, and Simpson’s index are employed here. The other compartment is called the equability index and shows the relative abundance of different species of a community in terms of their evenness of distribution. The evenness of a community depends on how close the number of individuals of different species is in the community. In the case of dominating species, the evenness index decreases. In order to compare alpha diversity results between groups, two statistical tests are used: F-test, for variance comparison, and the Mann-Whitney U test, to understand how similar the groups are.

Beta Diversity is estimated by the Bray-Curtis Dissimilarity Index (Equation (1)), which studies the mutual microbiota of two samples and the population in each sample [52]. The dissimilarity index returns with a value between 0 and 1; if both samples are identical the dissimilarity is returned as zero. In order to calculate the dissimilarity index between two samples, first, mutual species are selected, and then the less numerous species are summed to get *C_ij_* value. *S_i_* and *S_j_* given in Equation (1) represent the population of each sample.

(1) $${BC}_{ij}=1-\frac{{2C}_{ij}}{S_{i}+S_{j}}$$

Unweighted UniFrac Metric measures diversity in a qualitative manner and is based on the phylogenetic tree [53]. Multiple samples can be compared with this metric in a synchronized manner. Unweighted UniFrac metric uses branch length percentage to evaluate how far away communities are from each other.

Diversity calculations are done by using the microbiome package of R with raw abundance data retrieved from Aarts et al. [44] at the species level. In total, 96 samples and 98 species are used as input. The given CSV file is turned into the phyloseq format with the help of the phyloseq package.

### 2.4. Elasticities

The elasticity coefficient is used to analyze how sensitive exchange fluxes are to changes in exchange flux bounds or changes in bacterial abundance in the selected level. The elasticity coefficient formula (Equation (2)), implemented in the MICOM library, is used where *v* represents the exchange flux and *p* represents the parameter.

(2) $$\varepsilon_{p}^{v}=\frac{\partial \ln\left|v\right|}{\partial \ln\left|p\right|}$$

Flux direction is ignored with the absolute value but is defined separately to avoid information loss. The differentiation step size was selected as 0.1 on the log scale, resulting in a 10.5% increase in bacterial abundance in the native scale.

### 2.5. Probiotic Addition

*Lactobacillus rhamnosus* is selected as the probiotic provided to see how much it affects the selected metabolite fluxes and previously mentioned possible biomarkers. The amount of *L. rhamnosus* is determined with the least squares method, where the relative abundances of each ADHD sample’s gut microbiome are compared to a randomly selected Control sample’s abundance values. The abundance value of *Lactobacillus rhamnosus* to be added to the community model is calculated as the abundance, which gives the lowest difference between the Control sample and ADHD samples so that the gut microbiome of an ADHD subject becomes closer to the healthy one. In this study, the additional *L. rhamnosus* abundance is 0.2. After the probiotic addition, the same pipeline is followed for community model building and community growth in different media.

## 3. Results

### 3.1. Abundances

**Figure 2:**
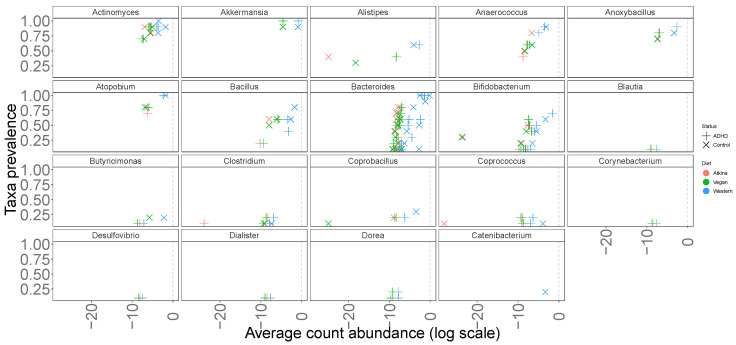
Taxa prevalence at Genus level.

Bacterial abundance data retrieved from Aarts et al. [44] are processed, and only those of matching pairs in terms of age and gender (Table 3) are here considered for analysis and gut community model building. The pairs (1 ADHD and 1 neurotypical Control at similar age) are used to compare microbial abundances and diversities in the gut. At the genus level, *Bifidobacterium* and *Bacteroides* have higher abundances in ADHD gut. Since the data of Aarts et al. [44] are used for Control and ADHD comparison, the abundance of the genus *Bifidobacterium* in ADHD gut is expected to be higher. At the species level, *Bifidobacterium adolescentis*, *Bifidobacterium dentium* and *Bifidobacterium pseudocatenulatum* have higher abundances in ADHD gut (Figure 2). In literature [54,55], *Bacteroides* genus shows lower abundance in ADHD gut, but *B. uniformis* has a higher abundance at species level in our study. *B. coprocola*, *B. plebeius*, and *B. vulgatus*, on the other hand, are found to have lower abundances in the guts of ADHD patients.

Most of the experimental studies on gut microbiome have reported their findings at the genus level. The genus *Parabacteroides* is expected to show a lower abundance in ADHD gut [56]. In our gut community model, the species *P. distasonis* and *P. merdae* show parallel behavior with literature findings, whereas *P. goldsteinii* has similar abundance values for both Control and ADHD cases. *Collinsella* genus is expected to be higher in ADHD gut [57]. At the species level, however, *C. aerofaciens* has lower abundance in most of the pairs and consequently in our gut community model. Like *Collinsella*, *Prevotella* is a possible biomarker at the genus level, and is expected to have a lower abundance in ADHD gut but a higher abundance in Control gut [56]. At species level, *P. buccae*, *P. copri*, and *P. ruminicola* satisfy this condition in the gut model. *Enterococcus* is a possible biomarker at genus level [54] and *E. faecium* is the only species passing the lower limit of abundance in our studied samples. Literature data show a lower abundance of Enterococcus in ADHD gut compared to Control samples. However, in the given data of Aarts et al. [44] abundance values are mostly similar for ADHD and Control cases. The same scenario applies to *Parabacteroides goldsteinii* and *P. xylaniphila* [58]; the latter has zero abundances for all pairs, and is therefore ignored in any future discussion.

Possible biomarkers of ADHD have been reported in the literature, but as these studies are conducted by different groups, these biomarkers are defined at different taxonomic levels (Table 4). For compatibility purposes, only samples at the species level are taken for data analysis and comparison.

### 3.2. Diversity

Diversities are used to characterize communities quantitatively by using the number of species and the number of individuals on any level, sample, habitat or community. There are three diversity measures: Alpha, Beta, and Gamma diversity. Alpha diversity shows the diversity in a habitat or community, and measures the richness and evenness of species in the habitat. Beta diversity shows diversity between different habitats or communities. Gamma diversity handles landscape level diversity [61]. Since the samples examined in this research are in the same landscape, gamma diversity will not be discussed here.

**Figure 3:**
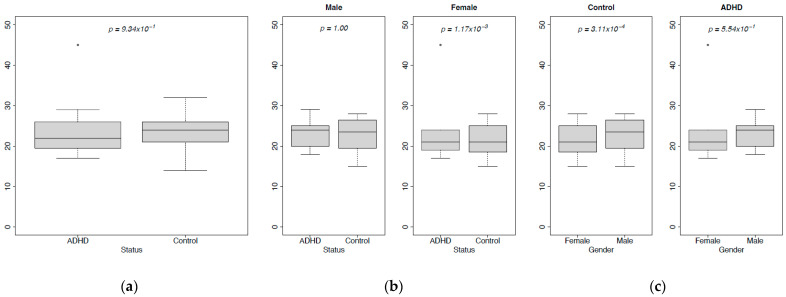

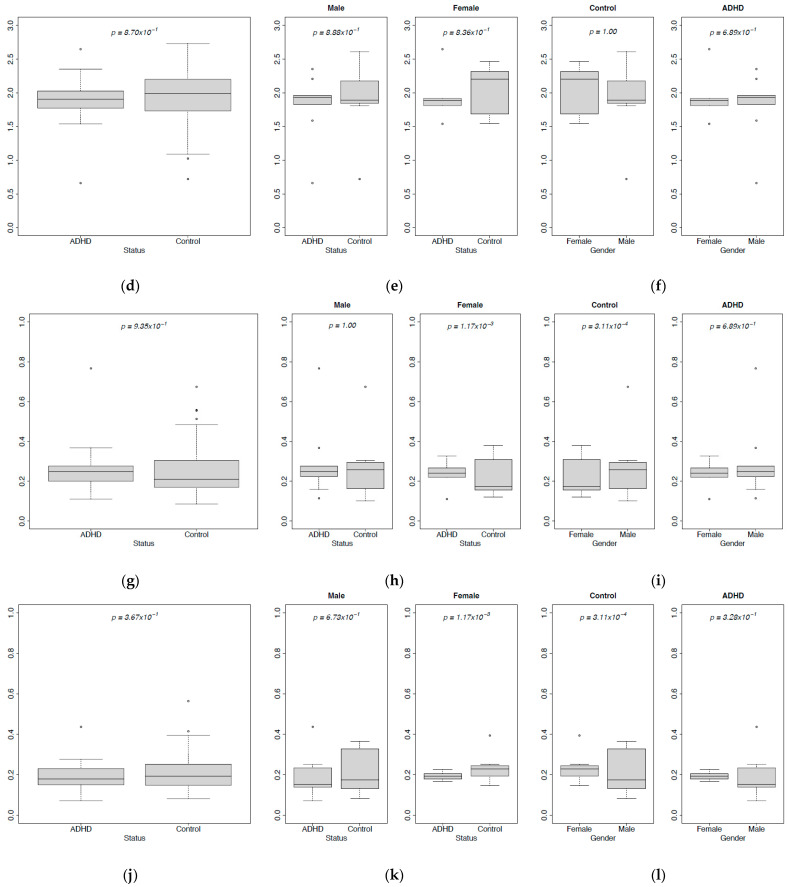
Distributions of alpha diversity metrics based on health status and gender: (**a**–**c**) Chao’s Index; (**d**–**f**) Shannon Index; (**g**–**i**) Simpson’s Dominance Index; (**j**–**l**) Evenness Index.

To characterize communities, alpha diversity is evaluated based on three indices: Chao, Shannon, and Simpson (Figure 3a–i). Chao index and Shannon index indicate that the Control gut has a higher median value than that of the ADHD gut. Even though both Control and ADHD samples have similar distributions in the number of species, the Control gut has a higher number of species than the ADHD gut (Figure 3a,d). Simpson index indicated that the deviation from the median for both groups (Control and ADHD) is almost equal, but they have significantly different mode values (the value that appears the most often in the data set) where ADHD subjects have a higher mode (Figure 3g). This means the number of dominant species is higher for the ADHD group compared to the Control group. There are some samples in the Control group with more dominant species, but there is no homogeneity. For all these three indexes, the Mann-Whitney U test does not find a significant difference between the median values of the number of species in the guts of Control and ADHD individuals.

For gender-based comparison, there are 17 male and 13 female samples; the gender of the rest of the samples is not known. The male group has eight Control and nine ADHD subjects while the female group has eight Control and six ADHD subjects. Corresponding to the variance difference, the Mann-Whitney U test does not find a significant difference between Control and ADHD subjects’ median values in either male or female populations (Figure 3b,e,h). Gender-specified data is not enough to make compelling guesses. However, Shannon’s index is more reliable than Chao’s index (in terms of gender-based comparison of Control and ADHD groups), since it is not dependent on sample size. Mann-Whitney U test finds a difference between median values of Control and ADHD groups for female subjects but not for males (Figure 3e).

In status-based comparison (different genders in the same status), there is no significant difference between the diversity values (Chao, Shannon, Simpson) of different genders in the same status. Mann-Whitney U test does not find any difference between male and female groups’ species richness (Figure 3c,f,i). Simpson’s index indicated that male Control subjects have a higher median value, but a lower mode value. This can be evaluated thus: the male Control group has lower dominance, but it is more homogeneous compared to female Control subjects under the same status (Figure 3i). The female ADHD group has a higher mode value, indicating a higher dominance in female ADHD subjects compared to males.

Evenness is a measure of equality. The value of evenness of a sample refers to the closeness of the number of individuals belonging to a species. Simpson’s Evenness Index is only used for this evaluation (Figure 3j–l). The Mann-Whitney U test gives the same results for status-based comparison, i.e., there is a location shift in both Control and ADHD populations. In the Control population, the female group has a higher median value and more symmetrical distribution. However, the male group has a higher mode compared to the female group. This can be interpreted as meaning that evenness in the male Control group is higher, but distribution is not homogeneous compared to the female Control group. In the case of the ADHD population, the female group has both higher mode and median values compared to the male group. The differences are not as significant as in the Control population, but they show a higher evenness for the female group.

**Figure 4:**
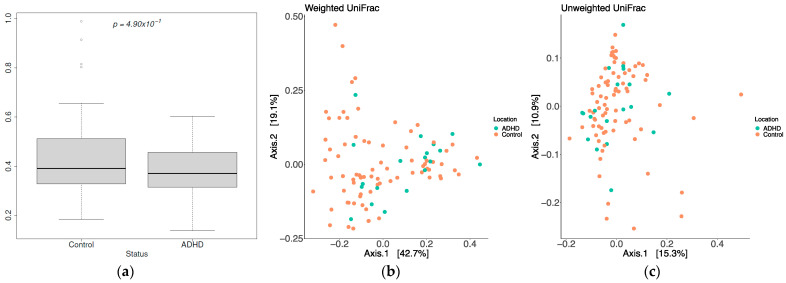
Beta diversity indices: (**a**) Bray-Curtis dissimilarity index results for Control and ADHD samples; (**b**) Weighted UniFrac distance results for Control and ADHD samples; (**c**) Unweighted UniFrac distance results for Control and ADHD samples.

To understand the differences between the two samples, Beta Diversity is employed with three metrics: Bray-Curtis Dissimilarity Index, Unweighted UniFrac Metric, and Weighted UniFrac Metric. Bray-Curtis’s dissimilarity index studies the mutual microbiota of two samples and the population in each sample [53]. The downside of the Bray-Curtis index is that the results depend on the sample population size. If the samples taken into account have small populations, small differences in species populations can have a significant effect on the dissimilarity index value. Although Control subjects have a higher variance (Figure 4a) in the BrayCurtis Dissimilarity test, there is no significant difference between the two environments (ADHD and Control) according to the Mann-Whitney U test (*p_mwu_* = 0.49). The Unweighted UniFrac Metric shows how different families can adapt to specific living conditions and how their adaptation differs. In Figure 4b, each point represents a sample of the population. Blue points belong to ADHD gut environment and orange points show subjects with a healthy gut. There is no significant difference or similarity between the two environments. The results do not provide conclusive discrimination between the two environments. Weighted UniFrac [62] is derived from Unweighted UniFrac as a quantitative metric, and it allows us to understand the effects of the relative abundance of species in a sample on the population. Weighted UniFrac gives better results compared to Unweighted UniFrac but, the results are still inconclusive (Figure 4c).

Although the diversity metrics use different assumptions and formulas for calculations and are affected by changes in the sample (such as changes in species composition, adding or subtracting species to the community, and dominant or hidden species), there are no significant differences in the results of the indices used to calculate alpha and beta diversities.

### 3.3. Production of Short-Chain Fatty Acids and Minor Metabolites (Amino Acids) by Gut Microbiome

Three different diets are applied to the reconstructed gut community model: Western, Atkins’, and Vegan. Western Diet is directly taken from the literature and represents the average American diet. The other two diets are curated from virtual metabolic human data [63]. Atkins’ and keto are alike in the sense of both being low-carb diets, but they have some minor differences in terms of their protein content. The ketogenic diet is a very lowcarb, moderate protein, high-fat diet plan. Atkins’ allows for a wider variety of foods, such as more fruits and vegetables and some grains. Thus, the Atkins’ Diet is a less restrictive approach.

SCFA production can reduce intestinal inflammation and may help to improve metabolic health [46]. The phylum/genus/species contributions to each SCFA (acetate, butyrate, formate, propionate) production/consumption, as well as their exchanges with the environment, are examined. The correlations with the diets, age, gender and health status (ADHD vs. healthy) are investigated. The strain contributors to the key SCFAs are mainly by the genera *Bacteroides*, *Bifidobacterium*, *Coprococcus*, *Dialister*, *Collinsella*, *Dorea*, *Prevotella*, and *Ruminococcus*, and the species *Alistipes putredinis* and *Subdoligranulum variabile*.

We compared the flux rates of each metabolite produced and/or consumed in the gut of ADHD subjects with those in healthy humans by flux balance analysis. The following section summarizes our results with significant flux values obtained by the gut community model. *Alistipes putredinis* is one of the species coming to the front as important SCFA producers in this study. In addition to more significant acetate production in ADHD subjects, small amounts of isobutyrate, isovalerate, and hydrogen are also produced by *Alistipes putredinis*. For each sample where acetate is produced (ADHD and Control), the Western Diet gives the lowest acetate export flux while the Vegan Diet gives the highest. The propionate export fluxes are also significant in both ADHD and Control subjects.

Formate production of *Bacteroides coprocola* in the ADHD samples is approximately 10 times higher than that of the Control samples. Formate import fluxes are lower while export fluxes are higher in *B. uniformis* for ADHD samples. In *B. vulgatus*, ADHD samples export higher amounts of formate. The amount of acetate by *B. coprocola* exported by the ADHD sample is almost 30 times higher than the neurotypical Control sample. In *B. uniformis*, ADHD samples mostly import acetate while Control samples export it.

The genus *Bifidobacterium* arises some questions as there are contradictory findings in the literature. Acetate has a considerable exchange rate in *Bifidobacterium dentium*, which is found only in ADHD subjects in the present gut community model. There are lower acetate export fluxes for the ADHD samples in *Bifidobacterium pseudocatelunatum*. Acetate fluxes mostly show import behavior and all samples that produce lower amounts of acetate are healthy. Acetate import fluxes for ADHD samples are high. The amount of formate produced by *Bifidobacterium pseudocatelunatum* has one of the highest values in this study, and this bacterium does not consume formate, while export is the only action (no formate import). Our study shows high butyrate, acetate, and formate exchange fluxes (both import and export) by *Coprococcus catus*, but the flux values of propionate are low and thus not significant in both ADHD and Control subjects. Formate export is mostly done by ADHD samples, and they have a lower relative abundance compared to Control samples. There is no effect of diet (no correlation with diet) on flux values, but the Western Diet shows significantly lower flux values for both ADHD and control cases. Acetate exchange rates, both import and export fluxes, are higher, between pairs, for ADHD samples. *C. catus* consumes acetate and butyryl–coa to produce acetyl–coa and this process limits butyrate production in the gut since butyryl–coa is also used to synthesize butyrate. *C. catus* is expected to have a negative effect on butyrate production, since it competes against butyrate-producing organisms for the necessary intermediates. In *C. catus*, in general, ADHD samples export more butyrate with lower abundance values. In the case of high abundance, export fluxes are almost equal to controls. Butyrate exchange done by another *Coprococcus* species, *C. comes*, also shows significant flux values. Acetate import and export fluxes by *Coprococcus comes* are also significant. The Western Diet results in the highest import and lowest export fluxes for acetate exchange out of the three different diets.

In this study, *Collinsella aerofacians* was found in 18 samples out of 20. Acetate, formate, and H_2_ exchange fluxes are significant compared to the fluxes of other metabolites; however, none of these fluxes outshine each other or are secerned. The SCFAs produced by *C. aerofacians* show both import and export behavior. H_2_ is exported only (no import by this bacterium) but export fluxes are mostly low with a few exceptions.

Under the genus *Dialister*, the asaccharolytic bacterium *Dialister invisus* is found in more than half of the ADHD subjects. The only significant fluxes come from acetate exchange, and the acetate export rate is higher for the ADHD sample. This bacterium is usually found in oral flora and in endodontic and periodontal infections [64]; this might be the reason why we encounter *D. invisus* in a high number of samples, though no significant metabolite fluxes were observed in this bacterium. In short, ADHD subjects might encounter this bacterium because of another underlying condition in a different part of the body.

Formate and acetate exchange fluxes are also significant in *Dorea formigeneras*. Control samples are prone to import formate. Control samples that export formate have lower reaction rates. Acetate fluxes are higher than formate fluxes in *Dorea formigeneras*. Acetate is only exported by *D. formigeneras*. Control samples produce acetate at a higher rate. *D. formigeneras* is more abundant in Control samples. *D. longicatena* imports L-tryptophan and exports H2 but fluxes are below 1 mmol/gDCW h. H_2_ export is an expected outcome of *D. longicatena* based on the literature, but tryptophan consumption or indole production is not mentioned previously for the gut of ADHD subjects in the literature. Formate and acetate have high exchange fluxes in *D. longicatena*. Formate export is not observed, and it is only imported by this species. Samples from subjects in their twenties mostly show higher formate import fluxes for Control samples in pairs. Pairs of matching siblings below the age of 20 do not show a correlation with flux. High amounts of acetate are exported by the bacterium *D. longicatena* as expected, but the acetate flux does not show any relation with age, health status, or diet.

In this study, the species *Prevotella copri*, *Prevotella ruminicola*, and *Prevotella buccae* are expressed in gut samples. *Prevotella copri* is expressed in five samples, each belonging to a different pair, with only one of them being an ADHD subject. Acetate, formate, and propionate are exported by this species *Prevotella copri*, but propionate fluxes are not significant. Acetate export by *Prevotella copri* is higher than formate export, as reported in the literature. Acetate export flux is lowest under the Western Diet and highest under the Vegan Diet. Additionally, for the genus *Prevotella*, in ADHD subjects, propionate, formate and acetate are also produced by another species, *P. ruminicola*. Propionate export rates are significant. Both subjects, ADHD and Control, show the highest propionate exchange rate when under the Western Diet. ADHD subjects have lower export fluxes than the Control sample, but subjects are not at the same age nor have the same gender. Acetate exchange is bidirectional for *P. ruminicola* and exchange fluxes are mostly significant. ADHD samples produce a higher amount of acetate than the Control samples, but these two types of samples belong to different ages and genders.

Under the genus *Ruminococcus*, *Ruminococcus bromii* is expressed in 16 samples out of 20, and six of these samples are amongst those diagnosed with ADHD. Formate and acetate exchanges were observed. Formate exchange done by *R. bromii* is bidirectional and exchange fluxes are low. Acetate is not produced, but only consumed by *R. bromii*, and the fluxes are significantly high. In terms of gender, female ADHD samples show a higher acetate import rate compared to their Control pairs with one exception. There are not enough pairs to compare male samples. In this study, 11 samples (three pairs), six of them diagnosed with ADHD, show the species *R. albus*. Formate and acetate are exported with significant fluxes by *R. albus*. ADHD samples have a higher abundance of this bacterium, as well as higher export fluxes compared to their Control pairs. The difference between ADHD and Control samples is higher for male pairs. Additionally, export fluxes decrease under the influence of the Western Diet. The Atkins’ Diet provides the highest flux values, but these rates are very close to those obtained under the Vegan Diet. There is no visible correlation between flux rates and age. Acetate fluxes are also higher for ADHD samples in pairs. However, there is no correlation between acetate fluxes and dietary habits, age, or gender.

Our results showed a significant butyrate import and acetate export in ADHD subjects with *Subdoligranulum variabile*. ADHD samples import more butyrate than the Control sample, which is the opposite for some pairs. In terms of acetate export, ADHD subjects show the lowest flux under the Western Diet effect. *S. variabile* also imports acetate for ADHD cases and has the highest import flux under Western diet influence.

There are thousands of different species that live in the human gastrointestinal tract. Some species, e.g., those belonging to *Bacteroides* species, are common in the gut population, but some others show sample-specific abundance. The species that appeared in very few samples will be discussed in Supplementary File S1.

### 3.4. Production of Precursors of Key Neurotransmitters in the Gut

Recent research revealed that gut bacteria can produce several neuroactive substances, such as gamma-aminobutyric acid (GABA), serotonin, and catecholamines such as dopamine and norepinephrine, or the precursors of these key molecules, which have an impact on ADHD. Theories about the neuroscience of ADHD place much emphasis on abnormalities in the noradrenergic, dopaminergic, serotoninergic, and cholinergic pathways [65]. In the present study, the precursors of the neurotransmitters are produced by the gut microbiota of age-matched siblings. Thus, glutamate (precursor of GABA), tryptophan (precursor of serotonin) and phenylalanine and tyrosin (precursors of dopamine) are examined by our community model.

Bacteria and neural tissue follow the same biosynthetic pathway to produce GABA. Glutamate is hydroxylated to GABA in the neuron cytoplasm [66]. Glutamate, the GABA precursor, is exchanged by gut flora in the present gut community model. Among the other neurotransmitter precursors, glutamate results in significant flux values in several bacteria like *Alistipes putredinis*, *Bacteroides vulgatus*, *Bifidobacterium angulatum*, *Coprococcus catus*, *Subdoligranulum variabile*. Glutamate fluxes also show a correlation with gender or dietary habits. *Alistipes putredinis* does not produce glutamate; however, it consumes this metabolite at a significant rate. The highest consumption rates are observed under the Western Diet influence with one exception. The flux differences in male pairs are more drastic compared to those in female pairs. Moreover, *Bacillus licheniformis*, *Bacteroides vulgatus*, *Blautia hydrogenotrophica*, *Coprococcus catus*, and *Prevotella ruminicola* show higher glutamate import fluxes under the Western Diet influence, whereas *Finegoldia magna*, *Mobiluncus curtisii*, *Parabacteroides ditasonis*, *Parabacteroides goldsteinii*, and *Prevotella copri* give the lowest glutamate exchange rates (Western Diet). Except for *P. copri*, all the species import glutamate, and none of them have insignificant flux rates. Only *F. magna* has a low glutamate import rate compared to other species. Vegan and Atkins’ diets give very close exchange fluxes for *F. magna* species. Glutamate exchange held by *P. ruminicola* is bidirectional, and, while the Western Diet provides the highest import rate, Atkins’ diet gives the highest export rate. In the case of *Holdemania filiformis*, ADHD samples import more glutamate than the Control sample. *C. eutactus* only imports glutamate, and female pairs show high differences between ADHD and Control samples compared to male pairs. For *Prevotella copri*, ADHD and neurotypical Control samples show almost equal glutamate export fluxes. However, as the number of samples including these gut bacteria is very limited, the results should be considered with care, and not taken as conclusive for this kind of rarely-present bacteria.

ADHD samples show higher L-tryptophan (serotonin precursor) import fluxes by *A. putredinis* compared to the Control, and no L-tryptophan export exists. This might result from a tryptophan hydrolysis reaction where tryptophan is hydrolyzed to indole, pyruvate, and ammonium with tryptophanase catalysis. There is no correlation between L-tryptophan import and the three diet types. In terms of gender, the difference between Control and ADHD sample fluxes of tryptophan is more drastic for male pairs than for female pairs. This might partially explain why males show more clear ADHD symptoms than females. ADHD gut also imports L-tryptophan by *Bacteroides coprocola* at a significantly higher rate. The amount of L-tryptophan imported by ADHD samples by another *Bacteroides species*, *B. uniformis*, is more significant compared to Control samples. *D. longicatena* imports L-tryptophan, but fluxes are below 1 mmol/gDCW h. For *D. longicatena*, tryptophan consumption or indole production is not mentioned previously for the gut of ADHD subjects in the literature. Only one subject with ADHD (female) has *Bacillus licheniformis* (0.12% abundance). There is L-tryptophan import under the Western Diet and export under Atkins’ and Vegan diets by this species, but these fluxes are not significant in amount.

Tyrosine and phenylalanine are both dopamine and noradrenaline precursors [67,68]. Tyrosine is produced from phenylalanine hydrolysis and DOPA is produced from tyrosine hydrolysis. In the end, DOPA is decarboxylated to dopamine [69]. These two metabolites show similar behavior in our gut model. They both give insignificant exchange fluxes most of the time. At other times, fluxes are still low, but since they are above 1 mmol/gDCW h, they cannot be seen as insignificant. These metabolites are not produced in some species. Bidirectional phenylalanine exchange is observed in *A. putredinis*, but flux values are insignificant. Tyrosine exchange only occurs in the import direction but again flux values are very low. Although flux rates are insignificant, phenylalanine and tyrosine import are also observed for *Bifidobacterium adolescentis*, *Bifidobacterium pseudocatelunatum* and *Coprococcus eutactus*, and that is important for model validation. Tyrosine and phenylalanine are also consumed by *Subdoligranulum variabile*. Both have insignificant flux values (less than 1 mmol/gDCW h), but their consumption information is important. While tyrosine is only imported by this bacterium, phenylalanine shows bidirectional fluxes. *S. variabile* consumes and produces phenylalanine under the influence of the Atkins’ and Vegan diets, but there is only consumption under the Western Diet.

### 3.5. Elasticities

**Figure 5:**
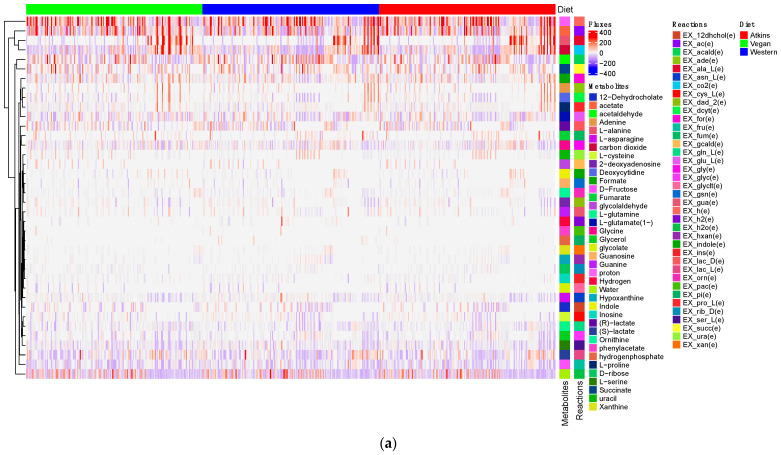

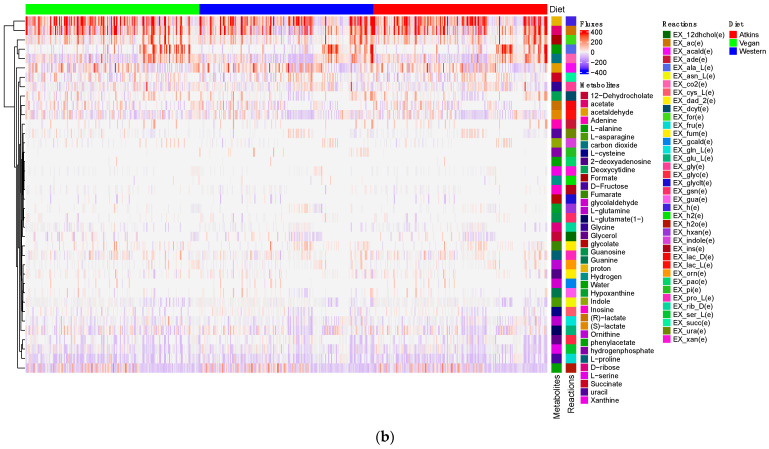
Metabolite exchange fluxes for (**a**) Control samples and (**b**) ADHD samples under Western, Atkins’ and Vegan diets.

The fluxes for each reaction in the gut community model were estimated for both ADHD and neurotypical Control cases by the computational pipeline embedded in MICOM, and those with relatively high values are given in Figure 5a,b, respectively. Elasticity coefficients are then used to analyze the sensitivity of bacterial abundances as well as exchange fluxes to changes in diets at the species level. The responses of the gut microbiota to three diets (Western, Atkins’, and Vegan) are summarized in Table 5, Table 6 and Table 7, where ADHD means a wider range of variances of elasticity coefficients for ADHD subjects, i.e., higher range of elasticities wrt healthy Control subjects. NA means that a bacterium is not present in that pair; same means the same range of variances of elasticity coefficients for both Control and ADHD subjects.

Under the influence of the Western Diet, *Bacteroides uniformis*, *Bacteroides vulgatus*, *Coprococcus catus*, and *Subdoligranulum variabile* consistently show higher elasticity variances in ADHD individuals (Table 5). *Alistipes putredinis*, *Collinsella aerofaciens*, *Coprococcus comes*, and *Subdoligranulum variabile* show higher elasticity variances for ADHD subjects under Atkins’ Diet (Table 6). Atkins’ Diet mimics the ketogenic diet. *Bacteroides uniformis*, *Bacteroides vulgatus*, *Bifidobacterium adolescentis*, and *Subdoligranulum variabile* have higher elasticity variances for ADHD subjects under the influence of the Vegan Diet (Table 7). Among the three diets, *Subdoligranulum variabile* is the common bacterium showing high elasticity variances, i.e., ADHD subjects’ metabolism is highly responsive to changes in this organism’s abundance.

Microbiota elasticity coefficients of the Atkins’ Diet have wider elasticity ranges in ADHD individuals. This diet also changes flux elasticity coefficients. The Atkins’ Diet affects the ADHD gut more than the Western Diet.

When the elasticity coefficients (and their variances) of exchange fluxes under all the diets are examined, lower elasticity coefficients are obtained in healthy individuals. It has been observed that the elasticity coefficients of the exchange fluxes of amino acid metabolism, minerals and metals such as zinc, cobalt, copper, oxygen and starch (carbon source of the microbiota) are high in ADHD individuals. The slightest change in these will seriously affect the intestinal system of ADHD individuals. In addition, the elasticity coefficients of chloride and proline in healthy individuals are found to be considerably lower than those in ADHD individuals, indicating a flux control. This result shows that the changes in the amounts of chloride and proline have less effect in healthy individuals, but there is no such trend in ADHD individuals.

In general, similar elasticity coefficients are obtained between healthy individuals and ADHD cases, but when the standard deviations are examined, lower values are calculated in healthy individuals, which is interpreted as healthy individuals being more resistant to changes and standing firm.

### 3.6. Intervention by a Probiotic (L. rhamnosus)

Probiotics are described as live microorganisms that provide the host with health benefits when given in sufficient doses. *Lactobacillus rhamnosus* (*L. rhamnosus*), which is sold as a nutritional supplement and added to a number of foods, including dairy products, is one of the most extensively researched friendly bacteria. *L. rhamnosus* promotes the growth of good bacteria, such as *Bacteroides*, *Clostridia*, and *Bifidobacteria*, in addition to preventing the colonization of harmful bacteria.

The exchange (import and export) behaviors of the short chain fatty acids such as acetate, formate, propionate and butyrate are investigated in detail by the gut community model, as *L. rhamnosus* helps increase their production. For example, upon probiotic addition to the community model, the ADHD sample starts to consume (import) butyrate at 4.90652 mmole/g DCW/h in *Subdoligranulum variabile* metabolism. Before the intervention, this particular male ADHD subject was exporting butyrate at a flux rate of 0.95877 mmole/g DCW/h under the Atkins’ Diet. Import rates of acetate and formate in *S. variabile* increase for most samples (ADHD and Control) for all diets after the *L. rhamnosus* addition. For *Bacteroides uniformis*, formate exchange fluxes show minor changes after the intervention. The pairs that export acetate show higher flux values for the sibling with ADHD. Moreover, the number of male samples importing acetate is higher than that of females. Similarly, *L. rhamnosus* addition to the environment affects the isobutyrate and isovalerate production ability of *B. vulgatus*. The acetate exchange carried out by *Bifidobacterium dentium* for ADHD samples is also impacted by the addition of *L. rhamnosus*. For *B. pseudocatelunatum*, after *L. rhamnosus* addition, acetate export flux values of ADHD subjects decrease by 5–15% under the Western Diet. The short-chain fatty acids produced by *Coprococcus catus*, acetate, butyrate, formate, and propionate, are affected significantly by the addition of *L. rhamnosus*. These changes in short-chain fatty acid fluxes varies by around 5–10% under the Atkins’ and Vegan diets for ADHD samples, but by 0–5% for Control samples under the Atkins’ diet and by 10–20% under the Vegan diet (mainly for acetate fluxes). Both Control and ADHD samples under the Western Diet show few cases with extreme changes. The other species under the genus *Coprococcus* is *C. comes*, which is also affected by probiotic intervention in terms of short-chain fatty acids exchange fluxes. Acetate shows the highest changes in flux values, which are decreased in both ADHD and control cases. All propionate flux values are increased but are still insignificant. On the other hand, *L. rhamnosus*’s addition to the system had no significant effect on *C. eutactus*.

Neurotransmitter precursors phenylalanine, glutamate, tryptophan, and tyrosine showed up, but the intervention did not result in any significant changes for these metabolites for any of the microbiota.

## 4. Discussion

The fact that people with ADHD frequently experience gastrointestinal (GI) dysfunction, such as constipation, low-grade inflammation, and childhood digestive problems, serves to highlight the possibility that the gut microbiome may play a role in this condition. Children with ADHD have been shown to have adverse reactions to particular meals and food additives, exposure to harmful dietary pollutants, and suboptimal levels of micronutrients such as vital fatty acids, zinc, magnesium, and iron. Food allergies can take the shape of hyperactivity. Given that ADHD children frequently exhibit a behavioral response to food, including real allergies, these two conditions may have similar pathophysiological routes [70]. Although it is likely that the gut microbiota plays a substantial role, there is rising evidence to show the role of nutrition in the regulation of ADHD behavior. Diet may thus contribute to the improvement of behavioral problems, probably through different unidentified complex mechanisms. It has long been known that the ketogenic diet has therapeutic benefits for children with autism spectrum disorder. Additionally, studies on animals have demonstrated that the ketogenic diet affects activity levels and trainability [71]. The effects of the Vegan Diet on ADHD or specific ADHD symptoms, such as inattention, hyperactivity, and impulsivity, have not been studied yet. However, there are some clinical studies [72].

Dietary interventions can be beneficial for children with ADHD. Thus, in the present study, the effects of the Western, Atkins’ (ketogenic) and Vegan diets on the prevalence of gut microbiota and their metabolites are investigated. In most of the cases examined in the present study, no strong correlation is found between the exchange direction of key metabolites (SCFAs and amino acids etc.) and health status, diets, gender, or age. Inferring with certainty whether a particular microbial profile is linked to ADHD is difficult given the results’ high degree of heterogeneity. The main findings on the key metabolites are summarized in Table 8.

In the present study, the species contributors to the key SCFAs mainly come from the phyla of Actinobacteria (genus *Bifidobacterium* and *Collinsella*), Bacteroidetes (genus *Bacteroides* and *Prevotella*), Bacillota (genus *Coprococcus* and *Subdoligranulum*), Bacteroidota (genus *Alistipes*), and Firmicutes (genus *Ruminococcus*, *Dialister*, and *Dorea*). *Alistipes* is a bile-resistant anaerobic acid-producing indole-positive genus [73]. The major product of *Alistipes putredinis* is succinate, as well as acetic, isobutyric, isovaleric, and propionic acids, which are produced in smaller amounts. *Alistipes* population found in the human gastrointestinal tract is correlated with fiber and processed food consumption in the diet [62]. Studies showed that the consumption of food of animal origin for a limited time increases the abundance of bile-tolerant and putrofactive microorganisms such as *A. putredinis* [74]. *Alistipes* genus is also related to fatigue and stress, which are also seen in mood disorders such as depression [75]. This might be related to *Alistipes* being an indole-positive organism that effects serotonin availability in a negative way [75].

One of the most dominant bacterial genera in the human gut is *Bacteroides*, with almost 25% of the bacteria in the human gut belonging to this genus [76]. *Bacteroides* species might pass from mother to child during birth and take their place in the gut microbiome from birth [77]. *Bacteroides coprocola*, *Bacteroides plebeius*, *Bacteroides uniformis*, and *Bacteroides vulgatus* are the species with a significant effect on samples studied here. *B. coprocola* is one of the L-tryptophan consumers and has been suggested to be an ADHD biomarker, since it showed lower abundance in the ADHD gut. *Bacteroides vulgatus* is another propionate-producing bacterium, and uses succinate as its main carbon source [78]. There is also evidence about the effects of *B. vulgatus* on cognitive function in Alzheimer’s patients via Glu metabolism [79]. *Bacteroides uniformis* is a probiotic used to modulate depression and anxiety in clinical studies done on rodents [80,81]. *B. uniformis* supplementation to mice affects reward response in the brain in a positive way and reduces anxiety [80].

*Bifidobacteria* is an immunoregulating bacteria genus [82]. *Bifidobacterium* species export low amounts of ethanol, formate, and succinate, but no butyrate or propionate production is possible. Tetrose and hexose phosphates are used to produce acetate. Some of the pyruvates can be used in acetate and formate production, which might alter the fermentation balance in the gut. Acetate import by *Bifidobacteria* is also possible, and acetate can be reduced to ethanol. There are four *Bifidobacterium* species with significant import and export fluxes, which are *Bifidobacterium adolescentis*, *Bifidobacterium angulaum*, *Bifidobacterium dentium*, and *Bifidobacterium pseudocatelunatum*. *B. adolescentis* is defined as a possible ADHD biomarker by a previous clinical study [57]. *Bifidobacterium adolescentis* in the human intestine has antidepressant effects. In the present study, there are nine pairs containing this species *B. adolescentis*, and ADHD abundance is higher for seven of them which contradicts the previous literature findings. *Bifidobacterium adolescentis* is another GABA-producing probiotic. An animal study proved that it has antidepressant effects and also reduces inflammation in the hippocampus [83]. In addition, ω − 3 fatty acid intake has an effect on the amount of *Bifidobacterium*, with the standard Western Diet having a 25:1 ratio of ω − 6 to ω − 3 fatty acids [84].

The genus *Coprococcus* is an anaerobic whose species are isolated from human feces [85]. *Coprococcus* uses carbohydrate fermentation as the main source of energy and growth [85]. Short-chain fatty acids—butyrate, acetate, formate, and propionate—are the main products of this genus. *Coprococcus catus*, *Coprococcus comes*, and *Coprococcus eutactus* are the species with significant metabolite fluxes. These species play a significant role in carbohydrate metabolism. *Coprococcus catus* is a known propionate producer which uses lactate as its main carbon source, and a study shows 3,4-dehydroxyphenylacetaldehyde to DOPAC conversion can be related to this species [86]. The conversion of 3,4-dehydroxyphenylacetaldehyde to DOPAC can be related to *Coprococcus comes*, in addition to *C. catus* [86].

*Dorea* uses glucose and some other sugars for acetate, formate, ethanol, H_2_, and CO_2_ production by fermentation. In addition to those metabolites, lactate production may be observed, but butyrate is not an expected output from this genus [87]. In this study, the species *Dorea formigeneras* and *Dorea longicatena* are observed. *D. formigeneras* is a formate-producing bacterium found in the human gastrointestinal tract. In order to produce formate, *D. formigeneras* follows a carbohydrate fermentation pathway. NaCl concentration in the environment may limit cell growth. As a result of glucose metabolism, *D. formigeneras* mainly produces acetate, formate, and lactate. The bacterium also utilizes pyruvate for acetate, formate, and ethanol production. Pyruvate utilization may result in lactate and succinate to a lesser extent. Propionate production from threonine is also possible. *D. longicatena* uses several carbon sources and mainly produces acetate, formate, and ethanol. Coexisting bacteria in the gut flora are able to use acetate, formate, and H_2_ produced by *D. longicatena*. In fact, this species is responsible for producing 10–30 g of acetate in the human gastrointestinal tract on a daily basis [87]. *D. longicatena* is a possible autism biomarker and the neurotransmitter precursor metabolites are not exchanged by this bacterium. Moreover, this species, *Dorea longicatena*, is only expressed in three ADHD samples.

*Prevotella* is one of the predominant genera in human gastrointestinal tracts. Some species of the genus are found in oral cavities [88]. In this study, *Prevotella copri*, *Prevotella ruminicola*, and *Prevotella buccae* were expressed in gut samples. *P. copri* is a predominant bacterium in human feces and isolated from it [89]. The bacterium uses several different sugars for acid production. The main products are acetate and succinate, but bigger fatty acid products also exist. *Bacteroides* in the growth medium might inhibit the growth of this species. Another species under the *Prevotella* genus is *P. ruminicola* which is mostly found in cow rumen [88]. Similar to *P. copri*, propionate, formate and acetate are produced by this species. *P. buccae* is a species isolated from human periodontal flora, human feces, and some other human tissues, and is not a bile-resistant bacterium [90]. The main metabolites produced by this species are acetate and succinate, but formate production is also observed for some strains. In terms of minor products, low isovalerate and isobutyrate exports occur.

*Ruminococcus* is a cellulolytic genus that lives in the mammalian gastrointestinal tract. Bacteria belonging to this genus require carbohydrates for growth. The main products of carbohydrate fermentation are acetate and formate; sometimes, succinate and ethanol might appear in the system [78]. In this study, *Ruminococcus bromii* and *Ruminococcus albus* were expressed in some samples. *R. bromii* is usually isolated from human feces and differs from other Ruminococcus species in terms of being non-cellulolytic. Instead of cellulose, *R. bromii* ferments starch [91].

*Subdoligranulum* species produce butyrate and lactate as major products of glucose and some other carbohydrates. *Subdoligranulum variabile* is a known butyrate producer living in human intestine and represents 1% of total *Subdoligranulum* population [92]. *Subdoligranulum variabile* also produces small amounts of acetate and succinate in addition to its main products [93].

Among the sample-specific species, *Akkermansia muciniphila* is an anaerobic bacteria found in the gut lumen of different mammals, including humans and rats, which uses the epital mucus layer as its carbon and nitrogen source [94]. *A. municiphila* is related to obesity, inflammation, Crohn’s disease, and insulin resistance [95], and is also found in gut samples of ASD children [25]. *Bacillus licheniformis* is a bacterium mostly found in bird feathers and soil [96]. It is responsible for β -keratin degradase in bird feathers [97]. Additionally, it is used as a probiotic for animals as an additive to animal feed [98,99]. Recently, *B. licheniformis* was isolated as a human probiotic [100,101], but there are not enough studies done in terms of probiotic effects on humans. *B. licheniformis* is mostly known for its effects on food. This bacterium is responsible for bread ropiness, food contamination, and the spoilage of food such as dairy products or bread [102,103]. *Blautia hydrogenotrophica* is a bacterium isolated from human and animal feces [104]. Its main purpose is H_2_/CO_2_ utilization for acetate production. In addition to acetate, *B. hydrogenotrophica* produces ethanol, lactate, and lower amounts of isobutyrate and isovalerate. Glucose and fructose are the main carbon sources for fermentation. *Clostridium paraputrificum* is a pathogen that causes several diseases in humans, such as bacteremia and myonecrosis [105]. A study showed that *C. paraputrificum* causes necrotizing cellulitis of the abdominal wall [106]. The bacterium utilizes glucose to produce H_2_ and CO_2_ [107]. The amount and composition of the end products are affected by the pH of the environment. *C. paraputrificum* also produces propionate and acetate as the main organic acid products, and the production of lactate and butyrate was also observed, but at lower flux values. The amount and type of organic acid produced affects the H_2_ yield rate. *Collinsella aerofacians* is a bacterium isolated from human feces. *Collinsella* genus produces ethanol, formate, H_2_, and lactate with fermentation. The main fermentation product of *C. aerofacians* is acetate and comes from sucrose and/or cellobiose, depending on which strain is isolated [108]. *Finegoldia magna* is an important pathogen found in the blood [109]. However, it can also be found in the gastrointestinal tract, female genito-urinary tract, and skin [110]. In fact, most of the *F. magna* cells used in in-vivo studies are isolated from skin, soft tissue, bone, or joint infections [110]. *Holdemania filiformis* is an acid-producing bacteria isolated from human feces [111]. It uses glucose as the main carbon source during acid production. Acetate, lactate, and succinate are major acid products of the bacterium. Formate might also show up as an additional acid product. *Mobiluncus* species are acid-producing bacteria. The main fermentation products of the genus are succinate and acetate, and lactate production may also occur. *M. curtisii* hydrolyses starch. Some strains of the bacterium were isolated from vaginal fluids taken from women with bacterial vaginosis diagnoses [112]. *Parabacteroides* is a bile-resistant genus commonly found in the human gastrointestinal tract [113]. Acetate and succinate are the major acids produced by this genus and other acids can be produced to a lower extent [113]. In this study, *P. distasonis*, *P. merdae*, and *P. goldsteinii* are expressed species of this genus. *P. distasonis* is a species isolated from human feces and uses several carbon sources to produce organic acids [114]. *P. merdae* also uses several carbon sources to produce organic acids. *P. goldsteinii* is another species isolated from human feces, whuch also uses several different sources of carbon for acid production [113]. In the presence of yeast and glucose, the bacterium is able to produce acetate and succinate. In addition to them, isovalerate, propionate, and formate can be seen to a lesser amount. As one can deduce from the abovementioned information, these sample-specific species might have been encountered in ADHD subjects as a result of a co-existing disease condition.

SCFAs affect numerous bodily functions, such as epithelial cell transport, metabolism, growth, and differentiation, as well as lipid and carbohydrate control in the liver. In addition to these, SCFAs also provide energy for organs such as kidneys, the heart, and the brain, tissues such as muscles, and epithelial cells at the end of the colon. Another function of SCFAs is to act as signaling molecules. For example, three SCFAs, mainly propionate and acetate, are ligands for G protein-coupled receptors of Gpr41 and Gpr43. These receptors are expressed at the end part of the small intestine, large intestine, and adipocytes.

SCFAs produced in the gut can enter the central nervous system by passing the blood–brain barrier, and glia and neurons consume these molecules. Consequently, SCFAs are a major energy source for glia and neurons. This cellular energy process is important for brain development, especially in the early years of life. In addition, SCFAs are used in the central nervous system for cell signaling, neurotransmitter synthesis, and release. SCFAs induce tyrosine hydroxylase, a catecholamine synthesis enzyme, and, as a result of this, dopamine and dopamine-related catecholamines production increase. Dopamine, serotonin, and glutamate systems in neurodevelopmental diseases suchas ASD are altered [115] and propionate has a similar effect on those systems.

The gut-brain axis is affected by microbially produced neurotransmitters like GABA or their precursors. Gamma-Aminobutyric Acid (GABA) is an inhibitory neurotransmitter and controls various physiological and psychological processes in brain. Anxiety and depression are related to GABA signalling problems. Glutamate decarboxylase (GAD) and vitamin co-factor pyridoxal phosphate are used to convert glutamate to GABA. Studies show that strains of *Lactobacilli* and *Bifidobacteria* can produce GABA from monosodium glutamate (MSG). *Lactobacillus brevis* and *Bifidobacterium dentium* (also present in our community model) are the most effective species for GABA production in MSG culture [32]. Fear and mood reactions are made to function properly via GABA_A_ and GABA_B_ receptors.

Serotonin regulates bodily functions such as mood, and is a product of tryptophan, an essential amino acid. In the present study, a comparison based on gender indicates some differences between the healthy and ADHD sample fluxes of tryptophan, which is more significant for male pairs than for female pairs (in the case of *Alistipes putredinis*). This might explain why males show more clear ADHD symptoms than females. Most commercial antidepressants focus on raising serotonin levels. Recent studies have shown that the serotonin level in regular mice is significantly high compared to that of germ-free mice, and this might be a result of a host–microbe interaction [116]. The effects of *Bifidobacterium infantis* on serotonin levels of germ-free mice were also observed and an increase in tryptophan in plasma was reported [117]. Tryptophan is a serotonin precursor and its increase shows that the bacterium has effects on tryptophan metabolism. In addition, lactic acid bacteria such as *Lactococcus lactis*, *Lactobacillus plantarum*, and *Streptococcus thermophilus* can also increase serotonin levels in plasma. However, these organisms are not observed in our gut community model due to their very low abundance in ADHD samples.

Dopamine and norepinephrine affect motor control, cognition, memory processing, emotion and endocrine regulation. Catecholamine neurotransmission dysregulation results in neurological and neuropsychiatric disorders such as Parkinson’s disease, Alzheimer’s disease, and major depression. Recent studies show that some bacteria can produce catecholamines. Dopamine is detected in *Bacillus cereus*, *Bacillus mycoides*, *Bacillus subtilis*, *Proteus vulgaris*, *Serratia marcescens*, *Serratia aureus*, and *Escherichia coli* biomass. In addition to the aforementioned species, lactic acid bacteria such as *Lactococcus lactis*, *Lactobacillus plantarum*, and *Streptococcus thermophilus* can produce dopamine. Norepinephrine was found in *Bacillus mycoides*, *Bacillus subtilis*, *Proteus vulgaris*, and *Serratia marcescens* biomass [36]. These organisms are lacking in our ADHD samples.

Some gut species show a direct relationship between the diet and metabolite exchange direction. For most species, Western Diet conditions cause an import behavior for dopamine precursors in the gut community model. In ADHD, the main problem with dopamine comes from the low number of dopamine receptors in the hippocampus. ADHD medications aim to produce more dopamine in the body to increase the number of molecules that find and bind to these receptors. The Western Diet restricts the precursor flux by forcing the bacteria to consume the precursors. The Atkins’ and Vegan diets, on the other hand, encourage the gut flora to produce these precursors.

Our computational study successfully revealed that the probiotic strain *Lactobacillus rhamnosus* (LGG) caused either increases in short chain fatty acid production fluxes or reversed their exchange direction (import/export) for some of the strains in the gut community model, in agreement with the available literature [54,65,118]. However, no effect of the probiotic intervention has been computationally observed on any neurotransmitter precursors. There are some recent reports that *Lactobacillus rhamnosus* GG significantly reduces the signs and symptoms of anxiety and sadness, with a preventive effect on ADHD, and also has a beneficial effect on cognitive function. The children and teenagers who received LGG supplements reported higher health-related quality of life [54,118]. Nevertheless, in light of the information gained from our computational study, as well as the very limited information in the literature, there is not enough evidence to strongly suggest the usage of probiotic supplements to treat ADHD today.

Each bacterium’s distinct genetic makeup results in the production of distinct sets of metabolites, which interact with host metabolites and additional compounds downstream to form a complex network of host–microbiome interactions. Our findings and those found in the literature suggest that medications that target the gut microbiota specifically may be effective in the treatment of ADHD. As the gut microbiota is an ecosystem, any alterations to one component would probably influence other parts. When designing such treatments, personalized medicine should be used due to the huge individual variation in the human gut microbiome.

By altering either the metabolic pathways or the expression of the genes encoding the neurotransmitter transporters, the gut microbiome may affect the catecholaminergic neurotransmission system [23]. Early in life, *Bifidobacterium* predominates in the gut and, as people mature, its relative abundance slowly declines. Thus, the fact that its abundance was lower in early infancy and rose in early adulthood may be a reflection of delayed gut microbiota maturation in ADHD. An increase in *Bifidobacterium* species was linked to significantly higher projected dopamine precursor (phenylalanine) biosynthesis potential in the gut microbiota of ADHD patients compared to controls [44,119,120].

The presence of Bacillota (genus *Coprococcus* and *Subdoligranulum*), Actinobacteria (genus *Collinsella*), Bacteroidetes (genus *Bacteroides*), Bacillota (genus *Coprococcus* and *Subdoligranulum*), and Bacteroidota (genus *Alistipes*) may be possible gut microbiota indicators of ADHD, as our case-control study is shown. *Alistipes putredinis* is especially associated with depression in the literature. Its consumption of high amounts of L-tryptophan is an unexpected behavior for ADHD subjects. In fact, L-tryptophan is a necessary metabolite for serotonin production, and higher consumption in people diagnosed with ADHD may help explain the relationship between depression and ADHD as well as ADHD and low serotonin. However, surely, a single bacterial genus cannot be directly responsible for any neuropsychiatric disorder, but rather they are the product of a complex interaction between numerous bacterial genera. Furthermore, the gut microbiome is not constant, and both internal and external factors can affect how the host responds. Researchers try to find explanations for the inconsistent findings already reported in the literature, and variations in the categorization and diagnosis of these neuropsychiatric illnesses also add to this problem, being another source of confusion in much research. Numerous investigations are likely to be complicated by the heterogeneity in these neuropsychiatric illnesses.

## Figures and Tables

**Table 2:** Age and gender-matched sub-samples from Aarts et al. [44].

Sample ID	Age	Gender
CONTROL01	15	Female
ADHD01	15	Female
CONTROL02	17	Female
ADHD02	17	Female
CONTROL03	18	Female
ADHD03	18	Female
CONTROL04	20	Male
ADHD04	20	Male
CONTROL05	20	Male
ADHD05	20	Male
CONTROL06	20	Female
ADHD06	20	Female
CONTROL07	21	Male
ADHD07	21	Male
CONTROL08	21	Male
ADHD08	21	Male
CONTROL09	22	Male
ADHD09	22	Male
CONTROL10	23	Male
ADHD10	23	Male

**Table 3:** Higher abundances in Control and ADHD Pairs.

Organism	Pair 1	Pair 2	Pair 3	Pair 4	Pair 5	Pair 6	Pair 7	Pair 8	Pair 9	Pair 10
*Bifidobacterium adolescentis*	Control	ADHD	Control	ADHD	ADHD	Same	ADHD	ADHD	Control	ADHD
*Collinsella aerofaciens*	Control	ADHD	Control	Control	Control	Same	ADHD	Control	Control	ADHD
*Bifidobacterium angulatum*	Control	ADHD	Control	Same	Control	Same	Control	ADHD	ADHD	Control
*Prevotella buccae*	Same	Same	Same	Control	Same	Same	Same	Same	Same	Same
*Prevotella copri*	Control	Control	ADHD	Same	Same	Same	Same	Control	Same	Control
*Bacteroides coprocola*	ADHD	Same	Same	Same	Same	Same	Control	Control	Same	Control
*Bifidobacterium dentium*	Same	Same	ADHD	Same	Same	Same	Same	ADHD	ADHD	ADHD
*Parabacteroides distasonis*	Same	Same	Control	Same	Same	Same	Same	Same	ADHD	Same
*Enterococcus faecium*	ADHD	Same	Same	Same	Same	Same	Same	Same	Same	Same
*Parabacteroides goldsteinii*	Same	Same	Same	Same	Same	Same	Same	Same	ADHD	Same
*Parabacteroides merdae*	Control	Control	ADHD	Control	Control	Control	ADHD	Control	ADHD	Same
*Bacteroides plebeius*	ADHD	Same	Same	Same	Same	Same	Control	Same	Same	ADHD
*Bifidobacterium pseudocatenulatum*	Control	ADHD	Control	ADHD	Control	ADHD	Control	ADHD	ADHD	Control
*Prevotella ruminicola*	ADHD	Same	Same	Same	Same	Same	Same	Control	Control	Same
*Bacteroides thetaiotaomicron*	Same	Same	Same	Same	Same	Same	Same	Same	Same	Same
*Bacteroides uniformis*	Control	Control	ADHD	Control	ADHD	Control	ADHD	ADHD	Control	ADHD
*Bacteroides vulgatus*	Control	Control	ADHD	Control	Same	Control	ADHD	Control	Control	ADHD
*Paraprevotella xylaniphila*	Same	Same	Same	Same	Same	Same	Same	Same	Same	Same

**Table 4:** Gut microbiota compositions in ADHD patients compared to Control groups.

Phylum	Family	Genus	Species	Higher in ADHD	Lower in ADHD
Actinobacteria					[57]
	Bifidobacteriaceae	*Bifidobacterium*		[44]	[54]
			*B. longum*		[57]
			*B. adolescentis*		[57]
	Coriobacteriaceae	*Collinsella*		[57]	
Bacteroidetes	Bacteroidaceae			[56]	
		*Bacteroides*			[54]
			*B. uniformis*	[55]	
			*B. ovatus*	[55]	
			*B. caccae*	[58]	
			*B. coprocola*		[55]
	Prevotellaceae				[56]
		*Prevotella*			[56]
		*Pararevotella*	*P. xylaniphila*	[58]	
	Porphyromonadaceae				[56]
		*Parabacteroides*			[56]
	Odoribacteraceae			[58]	
		*Odoribacter*	*O. splanchnicus*	[58]	
Firmicutes				[59]	[44]
	Catabacteriaceae				[56]
	Clostridiaceae	*Clostridium*	*C. histolyticum*		[54]
	Enterococcaceae			[58]	
		*Enterococcus*			[54]
	Lachnospiraceae				[58]
		*Lactobacillus*			[54,55]
	Ruminococcaceae				[58]
		*Faecalibacterium*			[58,60]
			*F. prausnitzii*		[58]
		*Ruminococcus*	*R. gnavus*		[58]
	Veillonellaceae	*Veillonella*			[58]
			*V. parvula*	[58]	
Proteobacteria	Neisseriaceae			[56]	
		*Neisseria*		[56]	
	Desulfovibrionaceae	*Desulfovibrio*		[59]	
	Sutterellaceae	*Sutterella*	*S. stercoricanis*	[55]	
Fusobacteria				[55]	
	Fusobacteriaceae	*Fusobacterium*		[55]	

**Table 5:** Elasticity comparison for Western Diet.

Organism	Pair 1	Pair 2	Pair 3	Pair 4	Pair 5	Pair 6	Pair 7	Pair 8	Pair 9	Pair 10
*Alistipes putredinis*	ADHD	Control	Control	Control	NA	NA	ADHD	Control	Control	Control
*Bacteroides coprocola*	NA	NA	NA	NA	NA	NA	NA	ADHD	NA	NA
*Bacteroides uniformis*	Control	ADHD	ADHD	ADHD	ADHD	NA	ADHD	Same	Same	Control
*Bacteroides vulgatus*	ADHD	Same	ADHD	Control	NA	Control	ADHD	ADHD	Control	ADHD
*Bifidobacterium adolescentis*	Control	Control	Same	Control	Same	NA	Control	ADHD	Control	Control
*Bifidobacterium angulatum*	ADHD	NA	NA	NA	NA	NA	NA	ADHD	NA	Control
*Bifidobacterium pseudocatenulatum*	ADHD	NA	NA	NA	NA	NA	NA	ADHD	Control	Same
*Collinsella aerofaciens*	Control	Control	Same	ADHD	ADHD	NA	Control	Control	Control	ADHD
*Coprococcus catus*	Same	ADHD	ADHD	ADHD	NA	NA	ADHD	ADHD	NA	Control
*Coprococcus comes*	Control	ADHD	Control	Control	ADHD	Control	ADHD	ADHD	Control	Control
*Coprococcus eutactus*	NA	NA	ADHD	ADHD	NA	NA	NA	Control	NA	NA
*Dialister invisus*	Control	NA	NA	ADHD	NA	NA	NA	NA	NA	Control
*Dorea formicigenerans*	Control	NA	Same	Control	Control	ADHD	ADHD	Control	ADHD	Control
*Dorea longicatena*	Control	ADHD	ADHD	Control	ADHD		Control	Same	Same	ADHD
*Holdemania filiformis*	NA	NA	NA	ADHD	NA	NA	NA	NA	NA	NA
*Parabacteroides merdae*	Control	ADHD	ADHD	NA	NA	NA	Control	NA	NA	NA
*Ruminococcus albus*	Control	NA	NA	ADHD	NA	NA	NA	ADHD	NA	NA
*Ruminococcus bromii*	Control	ADHD	NA	ADHD	NA	ADHD	ADHD	NA	Control	NA
*Streptococcus mitis*	Control	NA	NA	NA	NA	NA	NA	NA	NA	NA
*Streptococcus parasanguinis*	Same	NA	NA	NA	NA	NA	NA	NA	NA	NA
*Streptococcus thermophilus*	Control	Same	NA	NA	Same	NA	NA	NA	Control	NA
*Subdoligranulum variabile*	ADHD	ADHD	Control	ADHD	ADHD	ADHD	Control	Same	Control	ADHD

**Table 6:** Elasticity comparison for Atkins’ Diet.

Organism	Pair 1	Pair 2	Pair 3	Pair 4	Pair 5	Pair 6	Pair 7	Pair 8	Pair 9	Pair 10
*Alistipes putredinis*	ADHD	ADHD	Control	ADHD	NA	NA	ADHD	Control	Control	ADHD
*Bacteroides coprocola*	NA	NA	NA	NA	NA	NA	NA	Control	NA	NA
*Bacteroides uniformis*	Control	ADHD	Control	ADHD	Control	NA	ADHD	Control	Control	Control
*Bacteroides vulgatus*	ADHD	Control	Control	ADHD	NA	Control	Control	Control	Control	ADHD
*Bifidobacterium adolescentis*	Control	ADHD	Control	Control	ADHD	NA	ADHD	Control	Control	ADHD
*Bifidobacterium angulatum*	Control	NA	NA	NA	NA	NA	NA	Control	NA	ADHD
*Bifidobacterium pseudocatenulatum*	Same	NA	NA	NA	NA	NA	NA	Control	Control	ADHD
*Collinsella aerofaciens*	ADHD	ADHD	ADHD	Same	ADHD	NA	ADHD	Control	ADHD	ADHD
*Coprococcus catus*	Same	Same	Same	Control	NA	NA	ADHD	Control	NA	ADHD
*Coprococcus comes*	ADHD	Control	ADHD	ADHD	ADHD	Control	ADHD	Control	Control	ADHD
*Coprococcus eutactus*	NA	NA	ADHD	Control	NA	NA	NA	Control	NA	NA
*Dialister invisus*	Control	NA	NA	ADHD	NA	NA	NA	NA	NA	Control
*Dorea formicigenerans*	ADHD	NA	Same	Control	ADHD	ADHD	ADHD	Control	Same	Control
*Dorea longicatena*	ADHD	ADHD	Control	Control	ADHD	NA	Control	Control	Control	ADHD
*Holdemania filiformis*	NA	NA	NA	Control	NA	NA	NA	NA	NA	NA
*Parabacteroides merdae*	Same	ADHD	ADHD	NA	NA	NA	ADHD	NA	NA	NA
*Ruminococcus albus*	Control	NA	NA	Control	NA	NA	NA	Control	NA	NA
*Ruminococcus bromii*	Control	ADHD	NA	Control	NA	Control	ADHD	NA	Control	NA
*Streptococcus mitis*	ADHD	NA	NA	NA	NA	NA	NA	NA	NA	NA
*Streptococcus parasanguinis*	ADHD	NA	NA	NA	NA	NA	NA	NA	NA	NA
*Streptococcus thermophilus*	Control	ADHD	NA	NA	ADHD	NA	NA	NA	Control	NA
*Subdoligranulum variabile*	ADHD	ADHD	ADHD	Same	ADHD	ADHD	ADHD	Control	Control	Control

**Table 7:** Elasticity comparison for Vegan Diet.

Organism	Pair 1	Pair 2	Pair 3	Pair 4	Pair 5	Pair 6	Pair 7	Pair 8	Pair 9	Pair 10
*Alistipes putredinis*	Control	Control	ADHD	ADHD	NA	NA	Control	Same	ADHD	ADHD
*Bacteroides coprocola*	NA	NA	NA	NA	NA	NA	NA	Control	NA	NA
*Bacteroides uniformis*	Control	Control	ADHD	ADHD	Control	NA	Same	ADHD	ADHD	ADHD
*Bacteroides vulgatus*	Control	Control	ADHD	ADHD	NA	ADHD	Control	ADHD	Control	ADHD
*Bifidobacterium adolescentis*	ADHD	ADHD	ADHD	Control	Control	NA	Control	ADHD	Control	ADHD
*Bifidobacterium angulatum*	Control	NA	NA	NA	NA	NA	NA	ADHD	NA	ADHD
*Bifidobacterium pseudocatenulatum*	Control	NA	NA	NA	NA	NA	NA	Control	Control	Control
*Collinsella aerofaciens*	Same	ADHD	Control	Control	ADHD	NA	Control	Control	Control	Control
*Coprococcus catus*	Control	Control	ADHD	ADHD	NA	NA	Control	ADHD	NA	Control
*Coprococcus comes*	Control	Same	ADHD	ADHD	ADHD	ADHD	Control	Control	Control	Same
*Coprococcus eutactus*	NA	NA	ADHD	Control	NA	NA	NA	Control	NA	Control
*Dialister invisus*	Control	NA	NA	ADHD	NA	NA	NA	NA	NA	NA
*Dorea formicigenerans*	Control	NA	Control	Control	ADHD	ADHD	Control	Control	Control	ADHD
*Dorea longicatena*	Control	Control	Control	Control	Control	NA	Control	Same	Control	Control
*Holdemania filiformis*	NA	NA	NA	ADHD	NA	NA	NA	NA	NA	NA
*Parabacteroides merdae*	Control	ADHD	Control	NA	NA	NA	ADHD	NA	NA	NA
*Ruminococcus albus*	Control	NA	NA	Control	NA	NA	NA	ADHD	NA	NA
*Ruminococcus bromii*	Control	ADHD	NA	Control	NA	Same	Control		Control	NA
*Streptococcus mitis*	Control	NA	NA	NA	NA	NA	NA	NA	NA	NA
*Streptococcus parasanguinis*	Control	NA	NA	NA	NA	NA	NA	NA	NA	NA
*Streptococcus thermophilus*	Control	Control	NA	NA	Control	NA	NA	NA	Control	NA
*Subdoligranulum variabile*	ADHD	ADHD	ADHD	Control	ADHD	Control	Control	Control	Same	ADHD

**Table 8:** The relationship between the taxa, diets, and key metabolites *.

Key Metabolite	Western Diet	Atkins’ Diet	Vegan Diet
Acetate	***A. putredinis*** **🠗** ***C. comes*** **🠗** *C. comes* ↑ ***P. copri*** **🠗** ***R. albus*** **🠗** ***P. goldsteinii*** **🠗** ***P. buccae*** **🠕**	***P. goldsteinii* 🠕** ***R. albus* 🠕**	***A. putredinis*** **🠕** ***P. copri*** **🠕** ***R. albus*** ↑
Formate	***C. catus*** **🠗** *C. catus* ↓ ***P. goldsteinii*** **🠗** ***P. buccae*** **🠕**	***P. goldsteinii*** **🠕**	
Propionate	***P. ruminicola*** **🠕**	***P. goldsteinii*** **🠕**	*P. merdae* ↑ ***P. merdae*** **🠕**
Glutamate	*A. Putredinis* **🠕** *B. licheniformis* **🠕** *B. vulgatus* **🠕** *B. hydrogenotrophica* **🠕** *C. catus* **🠕** *P. ruminicola* **🠕** *F. magna* ↓ *M. curtisii* ↓ *P. distasonis* ↓ *P. goldsteinii* ↓ ***F. magna*** **🠗** ***P. distasonis*** **🠗** ***P. coprii*** 🠗	***P. ruminicola*** **🠕** *B. hydrogenotrophica* ↓ *F. magna* ↑	*P. goldsteinii* ↑ ***P. copri*** **🠕**
L-Phenylalanine	***M. curtisii*** **🠕** *P. distasonis* ↓ *D. formigeneras*	***D. formigeneras***	***D. formigeneras***
L-Tryptophan	*B. uniformis* ↓ *B. licheniformis*	***B. licheniformis***	***B. licheniformis***
L-Tyrosine	*M. curtisii* ↑ *D. formigeneras*	***D. formigeneras***	***D. formigeneras***

* Export fluxes are shown in bold font. The upwards arrow represents high flux values provided by the taxon and the downwards arrow represents the opposite. Some bacteria show bidirectional metabolic fluxes (export and import), where the diet achieves the same effect in both directions. This is why some bacteria are shown twice under the same diet. Additionally, for some species, changes in the diet directly affected the flux direction (cases *B. licheniformis* and *D. formigeneras*); these species are shown in the table without an arrow.

## Data Availability

The data used in this study are publicly available in the article of [44].

## Supplementary Materials

The following supporting information can be downloaded at: https://www.mdpi.com/article/10.3390/metabo13050592/s1, Supplementary Material File S1. Figure S1. Taxa prevalence at the Species level.
