# Supplementary material for: Understanding the ADHD-Gut Axis by Metabolic Network Analysis
Source: Metabolites. 2023 Apr 26;13(5):592. doi: 10.3390/metabo13050592 (PMC10223614; doi:10.3390/metabo13050592)
Supplement: Supplementary file 1 [file metabolites-13-00592-s001.zip › metabolites-2320888-supplementary.pdf]

Supplementary File S1:

Short chain fatty acid production by sample specific Bacteria in ADHD gut In this section, the species that showed up in very few samples will be discussed. *Akkermansia muciniphila* is only present in one subject with ADHD (male with 0.15% abundance). Significant formate and acetate export is observed in the gut model of this bacterium. While formate export shows the highest flux under Western diet influence, acetate export flux is the lowest.

Only one subject with ADHD (female) has *Bacillus licheniformis* (0.12% abundance). There is L-tryptophan (serotonin precursor) import under the Western diet, and export under Atkins' and Vegan diets by this species, but these fluxes are not significant in amount. In terms of short-chain fatty acids; formate, butyrate, and propionate are all consumed by *Bacillus licheniformis*. The highest import rate belongs to propionate while the lowest belongs to butyrate. Although there is a significant acetate export, other species in this female sample's gut lumen consume a high amount of acetate. *B. licheniformis* causes infections such as ventriculitis, ophthalmitis, bacteremia, peritonitis, and endocarditis. The reason why we encountered this species in an ADHD subject might be a coexisting infection in the body.

B. hydrogenotrophica was found in a male subject with ADHD (0.21% abundance). There is a significant H_2 consumption and acetate production. Apart from H2/CO2 utilization, *B. hydrogenotrophica* takes place in the Wood-Ljungdahl pathway where formate is used to produce acetate. This explains the significant formate import done by this bacterium. An ADHD subject (female) shows *Clostridium paraputrificum* (0.12% relative abundance) in the gut which is not expected since this bacterium is not a common pathogen in the human gut. Significant acetate, butyrate, and formate exports are observed, but the propionate production rate is lower than other SCFAs. H_2 export also exists but the flux value is insignificant. Since organic acid production other than acetic acid has a negative effect on the H2 production rate, one might speculate that high butyrate and formate productions block H2 production. We might have encountered this species as a result of a coexisting disease condition caused by another bacterium.

In this study, *Finegoldia magna* showed up in a female sample with ADHD (0.12% relative abundance). There is significant acetate, butyrate, and formate exports observed for the three diets and there are no significant differences between the fluxes under these diets. Since *F. magna* utilizes glucose or fructose for acid formation, high organic acid export is an expected outcome for this species. Although the acetate export rate is low compared to those of other bacteria in the gut of this female ADHD subject, it is still significant.

Three male subjects (2 ADHD, 1 Control) in this study present the bacterium *Holdemania filiformis*. There is a significant acetate export observed for all three samples. Formate exchange flux values are also significant but, the preferred exchange behavior is import. One sample with ADHD imports formate under the Western

diet influence but exports it for other diet types. In this study, a female sample with ADHD shows the bacterium *Mobiluncus curtisii*. Significant acetate and formate exports were observed but no correlation was found between flux values and diet.

Although *Parabacteroides* are common species in the human intestine, in our study, *P. distasonis* is expressed only in two unrelated samples' gut flora. One of the samples is a female subject with ADHD, while the other is a male Control subject. Both of the samples export high amounts of acetate and formate, but the flux values of both acids do not change with the three types of diets used in this study. Two female pairs express higher propionate export fluxes by *Parabacteroides merdae* in the ADHD sample. There is only one ADHD sample able to produce propionate under Western diet influence. The Vegan diet shows higher propionate fluxes compared to Atkins' diet. Formate exchange fluxes by *P. merdae* are significantly higher compared to propionate fluxes, and formate exchange is bidirectional. Export flux values of SCFAs are higher than their import rates, but the import is the dominant flux direction in *P. merdae*. In this study, the bacterium *P. goldsteinii* (0.15% relative abundance) is also only available in one male ADHD subject's gastrointestinal flora and exports acetate, formate, isovalerate, and propionate. There is no SCFA import behavior observed from this species. Isovalerate and propionate export rates are low and can be considered as insignificant compared to high export fluxes of acetate and formate. Isovalerate production cannot take place under the Vegan diet influence whereas the Western diet shows the highest flux value. Propionate, however, cannot be produced under Western diet influence, whereas Atkins' diet shows the highest flux rate. Both metabolites show flux values under 1 mmol/gDCW h; in this study, those values are mostly considered insignificant. Formate fluxes are unexpectedly high and the lowest value is achieved under the Western diet influence while the highest production is observed under Atkins' diet. Acetate fluxes are almost twice the formate export flux values. Acetate flux values react to diets the same way formate does.

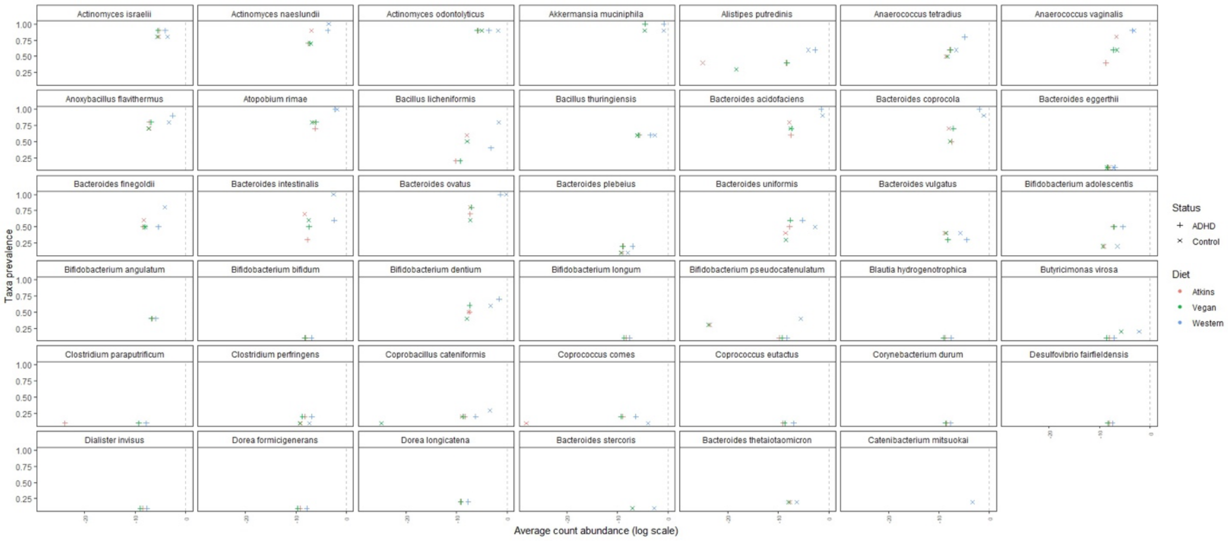

Supplementary Figure S1. Taxa prevalence at the Species level
